# Physical activity on prescription in patients with hip or knee osteoarthritis: A randomized controlled trial

**DOI:** 10.1177/02692155211008807

**Published:** 2021-04-11

**Authors:** Regina Bendrik, Lena V Kallings, Kristina Bröms, Wanlop Kunanusornchai, Margareta Emtner

**Affiliations:** 1 Department of Public Health and Caring Sciences/Family Medicine and Preventive Medicine, Uppsala University, Uppsala, Sweden; 2 Centre for Research and Development, Uppsala University/Region Gävleborg, Gävle, Sweden; 3 GIH, The Swedish School of Sport and Health Sciences, Stockholm, Sweden; 4 Faculty of Physical Therapy, Mahidol University, Bangkok, Thailand; 5 Department of Neuroscience, Uppsala University, Uppsala, Sweden; 6 Department of Medical Sciences, Uppsala University, Uppsala, Sweden

**Keywords:** Accelerometry, exercise therapy, osteoarthritis, physical activity

## Abstract

**Objective:**

To evaluate whether physical activity on prescription, comprising five sessions, was more effective in increasing physical activity than a one-hour advice session after six months.

**Design:**

Randomized, assessor-blinded, controlled trial.

**Setting:**

Primary care.

**Subjects:**

Patients with clinically verified osteoarthritis of the hip or knee who undertook less than 150 minute/week of moderate physical activity, and were aged 40–74 years.

**Interventions:**

The advice group (*n* = 69) received a one-hour session with individually tailored advice about physical activity. The physical activity on prescription group (*n* = 72) received individually tailored physical activity recommendations with written prescription, and four follow-ups during six months.

**Main measures:**

Patients were assessed at baseline and six months: physical activity (accelerometer, questionnaires); fitness (six-minute walk test, 30-second chair-stand test, maximal step-up test, one-leg rise test); pain after walking (VAS); symptoms (HOOS/KOOS); and health-related quality of life (EQ-5D).

**Results:**

One hundred four patients had knee osteoarthritis, 102 were women, and mean age was 60.3 ± 8.3 years. Pain after walking decreased significantly more in the prescription group, from VAS 31 ± 22 to 18 ± 23. There was no other between groups difference. Both groups increased self-reported activity minutes significantly, from 105 (95% CI 75–120) to 165 (95% CI 135–218) minute/week in the prescription group versus 75 (95% CI 75–105) to 150 (95% CI 120–225) in the advice group. Also symptoms and quality of life improved significantly in both groups.

**Conclusion:**

Individually tailored physical activity with written prescription and four follow-ups does not materially improve physical activity level more than advice about osteoarthritis and physical activity.

**Trial registration:**

ClinicalTrials.gov (NCT02387034).

## Introduction

It is well known that increasing the physical activity of people with osteoarthritis in the hip or knee is associated with a reduction in pain and an increase in mobility.^1–3^ The guidelines recommend 150 minutes per week of moderate or 75 minutes per week of vigorous physical activity,^4–6^ and those who do not meet these goals are particularly likely to benefit from more exercise.^1–3^ However, there is little guidance as how to increase physical activity in people with osteoarthritis.^7–9^ One review has shown that pain relief increases with the increased number of supervised exercise sessions,^10^ whereas others found no evidence that any one delivery mode worked better than others.^7–9^

Both European and international osteoarthritis guidelines suggest that physical activity interventions should be individualized to meet the patients’ needs and preferences.^5,6^ However, it is not clear whether a targeted personalized intervention could increase physical activity.^7–9^

In Sweden some patients are given a written prescription for exercise, known as physical activity on prescription, in an attempt to increase their physical activity. The physical activity on prescription method is based on a patient-centered dialog, individually tailored physical activity recommendations, a written prescription of the activities, and follow-up appointments.^11^ In patients with different risk factors, such as inadequate level of physical activity, impaired glucose tolerance, overweight, abdominal obesity, and surgery due to hip fracture, the physical activity on prescription method has shown positive results in increasing physical activity.^12^ This approach has not been evaluated in patients with hip or knee osteoarthritis. We hypothesize that physical activity on prescription is more effective in increasing physical activity than just providing advice about physical activity.

The primary aim of our study was to evaluate whether physical activity on prescription, comprising five sessions during the course of six months, was more effective in increasing physical activity than a one-hour advice session, in a population of patients with osteoarthritis of the hip or knee, after six months. Secondary aims were to evaluate the effects on fitness, symptoms, general health-related quality of life, and pain after walking.

## Method

This was a parallel group, assessor-blinded, randomized controlled trial of a six-month physical activity intervention with physical activity on prescription (prescription group) compared to advice (advice group). The study was conducted between June 2010 and August 2015. It was registered at ClinicalTrial.gov (NCT02387034) and was approved by the Regional Ethical Review Board, Uppsala (DNR2010/001). Region Gävleborg was the organization responsible for the integrity and conduct of the study. The project received funding’s from the Uppsala-Örebro Regional Research Council and from the Centre for Research and Development Uppsala University/Region Gävleborg. They had no influence on the research or the interpretation of the data.

The study was conducted in primary care at seven health care centers in a town in Sweden with 100,000 inhabitants. Patients who called or visited a nurse, physician, or physiotherapist seeking treatment for hip or knee pain, were told about the study. Patients interested in the study were screened by a physiotherapist at the same health care center and, if willing to participate, provided written informed consent.

Inclusion criteria were patients aged 40–74 years, with hip or knee pain and a verified clinical diagnosis of osteoarthritis,^13,14^ who self-reported less than 150 minutes of moderate or less than 75 minutes of vigorous physical activity per week. The physical activity level was assessed by interview using a validated questionnaire named Activity minutes^15,16^ (Supplement 1). Exclusion criteria were patients diagnosed with hip fracture or who had a history of hip or knee replacement, meniscal injury, cruciate ligament injury, neuropathic pain in the leg, rheumatoid arthritis, severe cardiovascular disease, or cancer. Those who could not communicate in Swedish were excluded.

**Figure 1.**
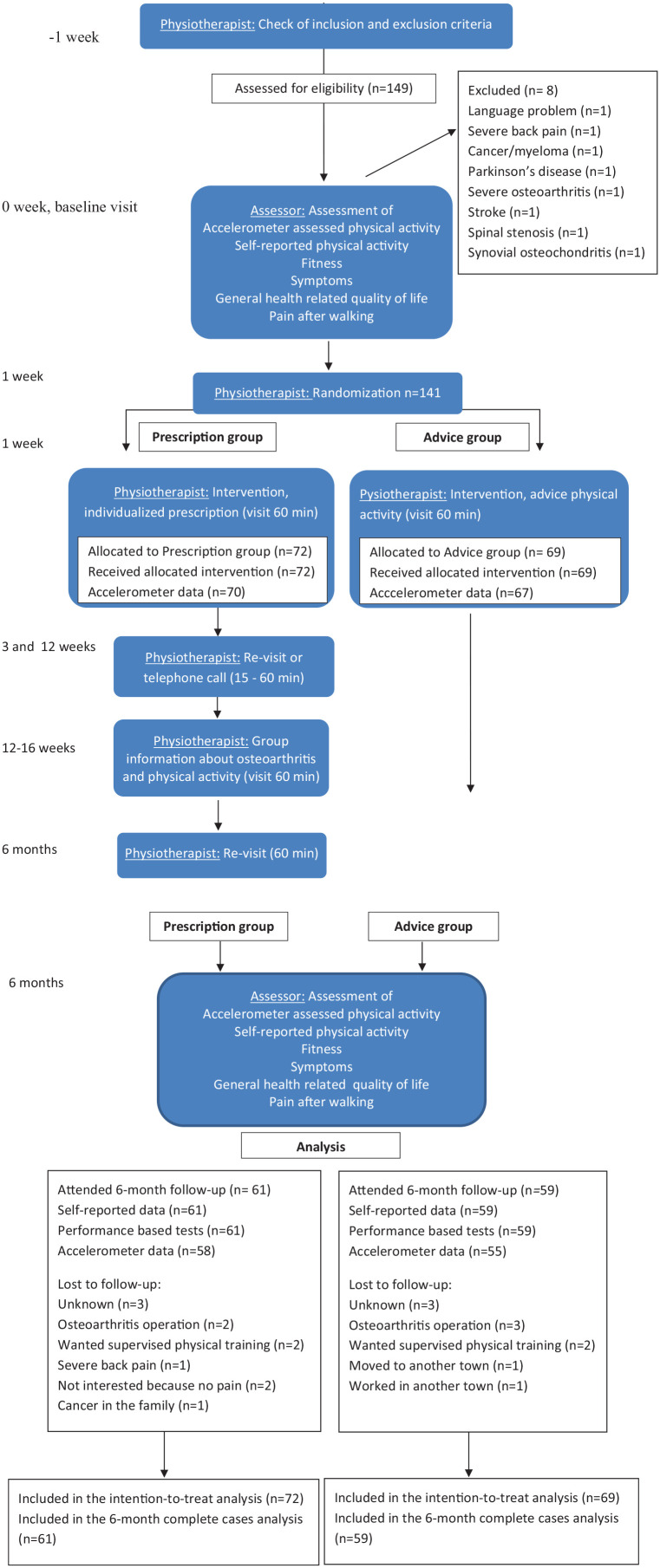
Flow of patients and analysis in the 6-month study period.

Block randomization was used. The assessor not involved in the study intervention generated the allocation. Sealed and opaque envelopes in groups of 10 (five each for prescription and advice groups) were prepared and distributed to each primary health care center. The physiotherapist opened the sealed envelopes to determine the groups. The assessor of outcomes performed all measurements. Participant characteristics, age, gender, education, employment status, location and duration of osteoarthritis, use of analgesics, comorbidity, and lifestyle habits (smoking, alcohol consumption, eating habits, and physical activity^15,16^) were assessed as part of the baseline questionnaire. Weight and height were measured and body mass index (BMI, kg/m²) calculated (Table 1). Primary and secondary outcomes were assessed at baseline and at six months (Figure 1):

- *Activity minutes*, two questions about exercise and everyday physical activity during an ordinary week. Activity minutes were summed from the two questions. The median value for each interval was used for calculating activity minutes for the individual (minutes in exercise × 2) + (minutes in every-day physical activity × 1) (Supplement 1).^15,16^
- *Leisure time physical activity* during the past year was assessed with one question with answers in four categories (sedentary, light physical activity, moderate exercise, and regular exercise (Supplement 1)).^17^
- *Sitting-time* was assessed with one question from the International Physical Activity Questionnaire (IPAQ) short form (Supplement 1).^18^
- *The six-minute walk test* to assess aerobic capacity.^19^ A minimal clinical change of ⩾ 14.0 m was evaluated.^20^
- *Visual Analog Scale* (VAS) (0–100 mm)^21^ to assess pain intensity after the six-minute walk test. A minimal clinical change of ⩾19 mm vas evaluated.^20^
- *Hip Disability and Osteoarthritis Outcome Score (HOOS)*^22^ and *Knee Injury and Osteo-arthritis Outcome Score (KOOS)*^23^ to assess joint-related symptoms. Each questionnaire includes five subscales (pain, other symptoms, activities of daily living, sport/recreation function, and quality of life). A minimal clinical change of ⩾10 points was evaluated.^23^
- *EuroQol (EQ-VAS* and *EQ-5D)* to assess general health-related quality of life.^24^ A minimal clinically improvement of ⩾ 0.08 in EQ-5D was evaluated.^25^
- The *30-second chair-stand test to* assess leg muscle strength.^19^ A minimal clinical change of ⩾2.0 repetitions was evaluated.^26^
- The *maximal step-up test* to assess muscle strength in each leg.^27^
- *The one-leg-rise test* to assess muscle strength in each leg.^28^
- *Accelerometer* to assess physical activity and sedentary time. A three-axis accelerometer with heath sensors, the SenseWear Armband Mini MF-SW (Body Media, Pittsburgh, Pennsylvania, USA) and the SenseWear Armband software 9 were used.^29,30^ The software calculates energy expenditure based on input data, in combination with the subjects’ height, weight, gender, and age, and presents the data in terms of steps taken and time spent at specific intensity levels. Sedentary time was defined as <1.5 metabolic equivalent of tasks (MET), light intensity physical activity as 1.5–2.9 MET, moderate and vigorous intensity physical activity as ⩾3 MET.^31^ Patients wore the sensor on the upper triceps 24 hours a day for seven consecutive days. All of the sedentary time minutes were added together from which a total sleep time of 450 minutes (7.5 hours) was subtracted. A valid day was counted as a day with 90% of 24 hours as wear time. Accelerometer data were eligible if the patient had worn the sensor at least four valid days. In order to identify and extract bouts of moderate and vigorous physical activity of 10 minutes or more, visual analyses of the data generated by SenseWear Professional 9.0 software were carried out for each valid day from all the participants.
- A seven-day diary supplemented the accelerometer data and helped to identify form, duration and intensity of physical activities.

The assessor, who collected and analyzed the data, was blinded to the patient’s allocation group. The physiotherapists were not blinded, since they had to treat according to the randomization. Patients were naturally not blinded to the interventions.

**Table 1.** Participant characteristics at baseline.

Characteristics	Randomized (*n* = 141)	
	Prescription group (*n* = 72)	Advice group (*n* = 69)
Women, *n* (%)	56 (78)	46 (67)
Age (years), mean (SD)	59.7 (8.6)	60.9 (7.9)
Body mass index (kg/m²), median (IQR)	31.0 (5.8)	30.2 (6.6)
Duration of symptoms (years), median (IQR)	2.0 (4.5)	1.5 (4.5)
Location OA		
Hip, *n* (%)	19 (26)	18 (26)
Knee, *n* (%)	53 (74)	51 (74)
Have used pain medication in the last week, *n* (%)	47 (65)	40 (58)
Comorbidity		
Depression, *n* (%)	6 (8)	5 (7)
Heart disease,^a^ *n* (%)	11 (15)	12 (17)
Asthma/COPD, *n* (%)	5 (7)	7 (10)
Severe obesity (body mass index (kg/m²) >35), *n* (%)	10 (14)	12 (17)
Severe pain (not due to knee or hip), *n* (%)	4 (6)	3 (4)
Diabetes mellitus, *n* (%)	3 (4)	4 (6)
Education		
Elementary school, *n* (%)	22 (31)	23 (33)
High school, *n* (%)	34 (47)	33 (48)
College/university, *n* (%)	16 (22)	13 (19)
Employment status^b^		
Working/studying, *n* (%)	39 (54)	39 (57)
Unemployed, *n* (%)	4 (6)	2 (3)
Sick leave, *n* (%)	4 (6)	5 (7)
Retired, *n* (%)	28 (41)	28 (39)
Lifestyle self-reported		
Current smoker, *n* (%)	6 (9)	7 (10)
Alcohol, risky consumption,^c^ *n* (%)	6 (8)	6 (9)
Eating habits, unhealthy eating,^d^ *n* (%)	7 (10)	9 (13)
Meeting 150 activity minutes/week,~ *n* (%)	19 (27)	14 (20)

a Heart disease: myocardial infarction, angina pectoris or heart failure.

b Employment status, participants can be in multiple categories.

c Alcohol, female risky consumption defined as: ⩾9 standard glasses/week or ⩾4 standard glasses on one occasion one or more times per months. For men defined as: ⩾14 standard glasses/week or >5 standard glasses on one occasion one or more times per months. A standard glass corresponds to 33 cl of beer, 12–15 cl of wine or just under 4 cl of hard liquor.

d Eating habits, unhealthy eating habits defined from a questionnaire index as: low consumption of fruit, vegetables and fish and high consumption of sweets, chips, buns and cakes, and soft drinks.

~ Numbers of patients meeting 150 activity minutes/week was calculated using the questionnaire activity minutes (Supplement 1).

Patients in the advice group received a session total of one-hour of information and individually tailored advice about physical activity (Figure 1). The information was both oral and printed, and comprised facts about osteoarthritis, physical activity, and weight control. The individually tailored physical activity included advice to practice: aerobic activities three times per week, activities they preferred, for example, walking or cycling, for at least 30-minutes; and muscle-strengthening activities in daily life, for example, to use stairs and focus on the legs when rising from a chair. There was no supervised physical activity from the investigators. The patients individually chose the duration and intensity of the activities.

The advice group was based on the behavior change techniques of information about health consequences and goal-setting (outcome goals).^32^ During the one-hour session, there was a discussion on potential harms and benefits associated with physical activity to enable the patient to make smart choices based on facts and not fears (information about health consequences). The goal-setting discussion resulted in one or several outcomes that the patient would like to reach (outcome goals).

Patients in the prescription group received the same one-hour session as the advice group, an additional written physical activity prescription, and thereafter four follow-up appointments. The prescription was based on what had been discussed and mutually decided on in the individual patient-centered dialog. It spelled out the type, form, frequency, and dose of physical activity, and provided a diary for self-monitoring of activities. Patients were not provided supervised physical activity by the investigators. At both three weeks and three months there was an individual follow-up either by telephone or in-person visit (15 minutes–1-hour). At three months there was a one-hour group booster session about osteoarthritis and physical activity, and at six months an individual one-hour follow-up visit.

The prescription group used additional behavior change techniques: goal-setting (behavioral goal), action-planning, self-monitoring of behavior, review of behavior goals, and graded tasks.^32^ During the first one-hour session the patient decided how to act to reach the goal (behavioral goal), and planned when, where and how the physical activities should be performed (action-planning). It could be both aerobic and muscle-strengthening activities, performed as home-based or supervised exercises, depending on the patient’s preference. They were also instructed to self-assess their behavior by writing down the daily activities in the diary (self-monitoring of behavior). At the individual follow-ups, the patient and physiotherapist evaluated the physical activity behavior and new goals and activities were planned or adjusted (review of behavior goals, graded tasks).

The required sample size was estimated with the purpose of securing sufficient statistical power for the analysis of the effects of physical activity measured as steps per day with accelerometer. Based on a previous similar study^33^ we assumed a mean difference of 600 steps per day between the prescription group and advice group in treatment effect, a standard deviation within-group of 1200 steps, and a correlation of 0.75 between assessments in the same person before and after the interventions. A two-tailed *t*-test on the difference in effect between the two groups was aimed to achieve a desired power of 80% (*P* = 0.05) if the sample size was approximately 70 patients per group.

Analysis followed the intention-to-treat principle and included all participants, including those with missing data and those who were not fully protocol compliant. If data were missing at the six-month follow-up, the patient’s own value from baseline was imputed, taken as last case forward.^34^ If data were missing at baseline, the patient’s own value from the six-month follow-up was imputed. Sensitivity analysis was performed, and data were analyzed separately as complete cases and compared with the results from intention-to-treat.

The change between-group was tested with the Welch *t*-test in normally distributed data and in case of skew outcomes, with the Mann–Whitney *U*-test. A difference in change for categorized outcomes between-group was tested with the chi-squared test. The change from baseline to follow-up within-group was tested using the paired *t*-test in numerical normally distributed data and using Wilcoxon’s signed-rank test in skew data. A difference in change for categorized outcomes within-group was tested with Friedman’s test. A two-sided *P*-value of less than 0.05 was set for statistical significance. Results are presented as between-group differences with 95% CI. All analyses were performed with the use of SPSS, version Statistics 22 (SPSS Inc., Chicago, IL, USA).

## Results

There were 149 patients assessed for eligibility. A total of 141 patients underwent randomization, 69 to the advice and 72 to the prescription group. Patient recruitment and reasons for loss to follow-up (*n* = 21, 15%) are shown in Figure 1. The six-month follow-up was attended by 59 and 61 patients from the advice and prescription groups, respectively. There were no adverse events reported in either group. Of the 141 participants, all had valid data in the fitness tests and in the questionnaires. Because of low wear time or technical problems, the accelerometer data were not valid in four patients at baseline (two in each group) and in seven patients at six months (four in the advice group and three in the prescription group) (Figure 1). Participant characteristics at baseline are presented in Table 1. Data from the intention-to-treat analyses are presented. Results from the intention-to-treat analyses did not differ significantly compared to the complete case analyses.

There were no between-group differences from baseline to six months in physical activity (Table 2). Self-reported activity minutes and leisure-time physical activity in both groups had improved at six months, whereas accelerometer-assessed physical activity remained stable in both groups (Table 2, Supplement 2). The most common activities were walking and cycling in both groups. Time in sedentary behavior, as measured with accelerometer and self-reported sitting-time, showed no between-group differences from baseline to six months (Table 2). There were no significant differences between groups in the six-minute walk test, in the 30-second chair-stand test, maximal step-up test, or one-leg-rise test from baseline to six months (Table 2). Pain intensity (VAS) after the six-minute walk test decreased significantly in the prescription group (Table 2). The six-minute walk distance met the standard for clinical improvement in the prescription group (⩾14 m)^20^ (Table 2).

**Table 2.** Comparison between-group for the variables physical activity, fitness, pain after the six-minute walk test, joint-related symptoms and quality of life, at baseline and after six months.

	Groups				Difference in change between-groups, *P*-value
	Baseline		6 months		
	Prescription group, *n* = 72	Advice group, *n* = 69	Prescription group, *n* = 72	Advice group, *n* = 69	
Self-reported physical activity	Median (95% CI) in activity minutes, mean (95% CI) in sitting-time				
Activity minutes, minutes/week	105 (75–120)	75 (75–105)	165 (135–218)*	150 (120–225)*	0.264
Sitting-time, hours/day	7.8 (7.1–8.5)	7.2 (6.6–7.9)	6.5 (5.9–7.1)*	6.1 (5.4–6.8)*	0.709
Accelerometer assessed physical activity	Median (95% CI)				
MVPA, ⩾10 minutes bouts, minutes/day	31 (22–43)	31 (26–37)	35 (27–36)	30 (30–40)	0.253
MVPA, minutes/day	70 (29–77)	62 (32–70)	69 (29–78)	60 (31–71)	0.821
Light PA, minutes/day	225 (203–252)	220 (190–232)	218 (195–247)	215 (205–221)	0.692
Sedentary, hours:minutes/day	10:52 (10:22–11:30)	11:20 (10:49–11:34)	10:40 (10:15–11:37)	11:20 (10:56–11:34)	0.704
Steps, number/day	7531 (6358–8589)	7161 (6940–7947)	7715 (6263–8477)	6972 (6267–7722)	0.505
METs, average/day	1.2 (1.1–1.2)	1.2 (1.1–1.2)	1.2 (1.2–1.2)	1.1 (1.1–1.2)	0.306
Total energy expenditure, kcal/day	2441 (2250–2552)	2404 (2246–2611)	2421 (2300–2540)	2398 (2258–2608)	0.777
Fitness and pain after six-minute walk test	Mean (95% CI)				
Six-minute walk test, (m)	501 (483–520)	510 (492–525)	521 (500–542)*	518 (498–536)	0.130
Pain intensity after six-minute walk test, VAS^e^	31 (26–36)	26 (21–31)	18 (13–23)*	23 (18–28)	0.016
30-second chair-stand test (*n*)	11 (10–12)	11 (11–12)	12 (11–13)*	12 (12–13)*	0.872
Maximal step-up test, (affected leg) (cm)	22.1 (20.7–23.8)	23.7 (22.0–25.4)	24.5 (23.1–26.0)*	25.3 (23.6–27.0)*	0.255
One-leg-rise test (affected leg) (*n*)	0 (0–0)	0 (0–0)	0 (0–0)	0 (0–0)	0.973
Joint-related symptoms HOOS/KOOS	Mean (95% CI)				
Pain	52 (48–56)	55 (51–58)	65 (60–69)*	65 (60–69)*	0.407
Other symptoms	57 (52–62)	57 (53–61)	65 (60–70)*	66 (61–71)*	0.738
Activities of daily living	61 (57–65)	62 (58–66)	70 (66–75)*	70 (66–75)*	0.655
Sports and recreation	31 (26–37)	31 (26–36)	41 (34–46)*	40 (34–46)*	0.887
Quality of life	39 (35–43)	39 (35–42)	51 (46–56)*	49 (44–54)*	0.501
General quality of life	Mean (95% CI)				
EQ-VAS	65 (61–69)	64 (59–68)	69 (65–73)	68 (63–72)*	0.881
EQ-5D	0.60 (0.55–0.66)	0.61 (0.55–0.66)	0.71 (0.65–0.75)*	0.71 (0.65–0.75)*	0.840

CI: confidence interval; MVPA: moderate and vigorous physical activity (⩾3.0 METs); light PA: physical activity (1.5–2.99 METs); sedentary (<1.5 METs); VAS: visual analog scale 0–100 (0 = no pain, 100 = worst). 30-second chair-stand test measures the number of times an individual can go from sitting to standing to sitting and so on in 30 seconds, chair height 44 cm, as an objective assessment of lower-limb muscle strength. Maximal step-up test measures the patients’ maximal step-up height in 3 cm intervals in one leg without compensation from the other leg as an objective assessment of lower-limb muscle strength. One-leg-rise test measures the number of times an individual can go from sitting to standing, chair height 48 cm, as an objective assessment of the affected leg; HOOS: hip disability and osteoarthritis outcome score; KOOS: knee injury and osteoarthritis outcome score, ranges from 100 (best) to 0 (worst); EQ-VAS: EuroQol Group visual analogue scale, with scores ranging from 100 (best) to 0 (worst); EQ-5D: EuroQol Group-5D, the three level version of questionnaire, descriptive index with scores ranging from 1.00 (best) to 0 (death).

* *P*-value <0.05 from baseline to six months in group.

Pain, other symptoms, activities of daily living, sports/recreation function, and quality of life assessed with HOOS/KOOS, did not show any between-group differences from baseline to six months. In both groups, there were significant within-group improvements over baseline in all five subscales. The threshold for clinical improvement, defined as a result that improved ⩾10 points,^23^ was exceeded for the subscales pain and quality of life in both groups (Table 2). General health-related quality of life, assessed with EQ-5D, did not show any significant difference between-groups from baseline to six months, but met the standard for clinical improvement in EQ-5D (value ⩾ 0.08) in both groups (Table 2).

## Discussion

Our hypothesis was that the intervention, physical activity on prescription, should be more effective to increase physical activity compared to advice. We expected that a comprehensive intervention based on several behavior change techniques, with a written prescription, and several follow-up appointments should be effective in patients with osteoarthritis in the hip or knee.^11,12,35,36^ The result of this randomized controlled study showed that the physical activity on prescription, did not work sufficiently well to provide any benefit over advice. There were no significant differences between the groups in any outcome at six months except in pain after walking (VAS). However, this improvement could be a result of a higher level of pain at baseline, a regression to the mean, or multiple tests. Both groups improved over baseline in self-reported physical activity, fitness, symptoms, and quality of life.

Similar results in both groups might have been due to the fact that the groups were too similar. Patients in both groups were offered an individualized approach that promoted physical activities based on the patient’s needs and preferences. This individualized approach, as suggested in guidelines,^5,6^ was probably an important component. Additionally, the behavior change techniques “information about health consequences” and “goal-setting (outcome goal)” were used in both groups and probably contributed to the improvements.^35–37^ We speculate that by discussing the role of physical activity, its potential harms and benefits, and what the patients easily could do themselves influenced patients in both groups positively. Setting realistic and achievable goals leads to increased physical activity,^35–37^ whereas information alone has not been shown to change behavior.^36^ In both groups, patients were given support to perform physical activities in daily life, but they could also arrange on their own to take part in supervised exercise sessions if they chose. Thus, patients were given opportunities, in accordance with their needs, to choose how and when they could be physically active. Also to confirm that physical activities in daily life can be sufficient, is an important message. We had expected the prescription intervention, with several behavior change techniques, and structured follow-ups, to be more beneficial, but these extra efforts were probably not necessary.

There may have been other reasons why the advice group improved as much as it did. When they were invited to participate in the study and throughout baseline testing, the advice group may have become more aware of the benefits of physical activity and were thus more receptive to suggestions and more adherent to individualized advice from the physiotherapist. They might also have searched the internet for information about osteoarthritis and physical exercise. Aligning with our results a meta-analysis evaluating physical activity interventions in primary care found that briefer interventions were able to achieve effects that were similar to the more intensive ones.^38^

We used several behavior change techniques in the prescription group, which makes it impossible to distinguish the single most effective technique. Also in agreement with our study, a combination of self-monitoring and goal-setting was shown to result in improvements in pain and function in individuals with knee osteoarthritis^39^ and a systematic review of patients with lower-limb osteoarthritis found goal-setting, behavior contracts, self-monitoring of behavior, social support, and non-specific rewards to be effective in promoting physical activity.^35^ Thus, there is still only limited evidence as to which behavior change techniques are the most efficient in individuals with hip or knee osteoarthritis.^35^

Both groups significantly improved over baseline in self-reported physical activity, fitness, symptoms, and general quality of life. Although these improvements were clearly observed, it cannot be stated that the interventions themselves were beneficial, as there was no placebo group. Our advice group was the same as usual care in Sweden, therefore a placebo group in this sort of study would have been unethical. Though both our groups improved in terms of self-reported physical activity, improvements in accelerometer-assessed activity were not observed. Our results are in accordance with two meta-analyses in individuals with chronic musculoskeletal pain and in individuals with osteoarthritis in the hip or knee.^7,9^ One possible reason for the difference between accelerometer-assessed and self-reported physical activity might be that the accelerometer measures activity in absolute intensity, while self-reported questionnaires measure relative intensity. For example, a person with osteoarthritis may experience and self-report a short walk as vigorous activity (the relative amount of intensity), while the accelerometer captures the event as light activity (the absolute amount of intensity). Another reason is that accelerometers do not reliably detect activities such as cycling, swimming, and strength training.^40^

This study was the first to evaluate the Swedish method of physical activity on prescription in patients with osteoarthritis.^12^ Like other studies on physical activity on prescription, our patients increased self-reported physical activity^41–43^ but not accelerometer-assessed physical activity.^44,45^

The strength of our study was its focus on individualized treatments in both study groups, as recommended in guidelines.^5,6^ Another strength was the use of different behavior change techniques^32^ and the role of physiotherapists in standard primary care to perform the study. Multiple measures, such as accelerometer, fitness tests, and questionnaires, were used to capture physical changes.

Our study also had limitations. The two interventions were quite similar and there was no true control group. The study sample was relatively physically active at baseline with little room for major improvements. Finally, the dropout rate in accelerometer-assessed data was 20%, which could have resulted in a type-2 error. However, dropout rates were similar between groups and was lower (15%) in the questionnaires and fitness tests.

In conclusion, an individualized physical activity intervention according to the patient’s needs and preferences can be used in primary care to improve the level of physical activity, quality of life, and symptoms for patients with osteoarthritis of the hip or knee. Further research should evaluate which behavioral change technique is most beneficial and which patients benefit from which interventions.

**Clinical message**

- Individually tailored physical activity with written prescription and four follow-ups does not materially improve physical activity level more than individualized advice about osteoarthritis and physical activity.

## Supplemental Material

sj-pdf-1-cre-10.1177_02692155211008807 – Supplemental material for Physical activity on prescription in patients with hip or knee osteoarthritis: A randomized controlled trial Click here for additional data file. Supplemental material, sj-pdf-1-cre-10.1177_02692155211008807 for Physical activity on prescription in patients with hip or knee osteoarthritis: A randomized controlled trial by Regina Bendrik, Lena V Kallings, Kristina Bröms, Wanlop Kunanusornchai and Margareta Emtner in Clinical Rehabilitation
