# Supplementary material for: Physical activity on prescription in patients with hip or knee osteoarthritis: A randomized controlled trial
Source: Clin Rehabil. 2021 Apr 11;35(10):1465–77. doi: 10.1177/02692155211008807 (PMC8495310; doi:10.1177/02692155211008807)
Supplement: sj-pdf-1-cre-10.1177_02692155211008807 – Supplemental material for Physical activity on prescription in patients with hip or knee osteoarthritis: A randomized controlled trial [file sj-pdf-1-cre-10.1177_02692155211008807.pdf]

Supplement 1. Questionnaires, self-reported physical activity and sitting time.

Activity minutes were assessed by a questionnaire consisting of two questions about self-reported exercise and everyday physical activity during an ordinary week [15, 16].

*a) How much time do you spend during a typical week on **physical training** that makes you feel short of breath, for instance running, jogging, or ball games?*

- ☐ 0 minutes
- ☐ <30 minutes
- ☐ 30-60 minutes (0.5 -1 hour)
- ☐ 60-120 minutes (1-2 hour)
- ☐ >120 minutes (2 hour)

*b) How much time do you spend during a typical week for **daily physical activities** such as walking, cycling or gardening?*

- ☐ 0 minutes
- ☐ <30 minutes
- ☐ 30-60 minutes (0.5 – 1 hour)
- ☐ 60-90 minutes (1-1.5 hours)
- ☐ 90-150 minutes (1.5 – 2.5 hours)
- ☐ 150-300 minutes (2.5 – 5 hours)
- ☐ >300 minutes (5 hours)

Activity minutes were summed up from the two questions (minutes in physical training x 2) + (minutes in daily activities x 1) [15, 16]. The median value in the category was used.

Leisure time physical activity during the past year was assessed with one question with answers in four categories [17].

How much have you moved about and exerted yourself physically in your leisure time in the last 12 months? If your activity varies between e.g. summer and winter, try to take an average. Response options were as follows:

☐ Sedentary leisure time.
You spend most of your time reading, television, at the cinema or other sedentary activities in leisure time. You walk, cycle or move about in other ways less than 2 h a week.

☐ Light physical activity during leisure time.
You walk, ride a bicycle or move about in other ways, for at least 2 h a week without sweating. This includes e.g. walking or cycling to and from work, other walks, heavier household work, normal gardening, fishing, tennis or bowling.

☐ Moderate exercise during leisure time.
You exercise regularly 1–2 times per week for at least 30 min at a time, e.g. running, swimming, tennis, badminton or other activity that makes you sweat.

☐ Regular exercise and training.
You participate in for example running, swimming, tennis, badminton, exercise gymnastics or similar activities that makes you sweat at least 3 times per week. Each session lasts at least 30 min at a time.

Self-reported sitting-time was assessed with one question from the International Physical Activity Questionnaire (IPAQ) short form [18].

How much time (hours) per day do you spend sitting on average during a weekday? Try to estimate, for example, the time sitting during work, studies, leisure, eating, talking and watching TV.

________________ hours per day

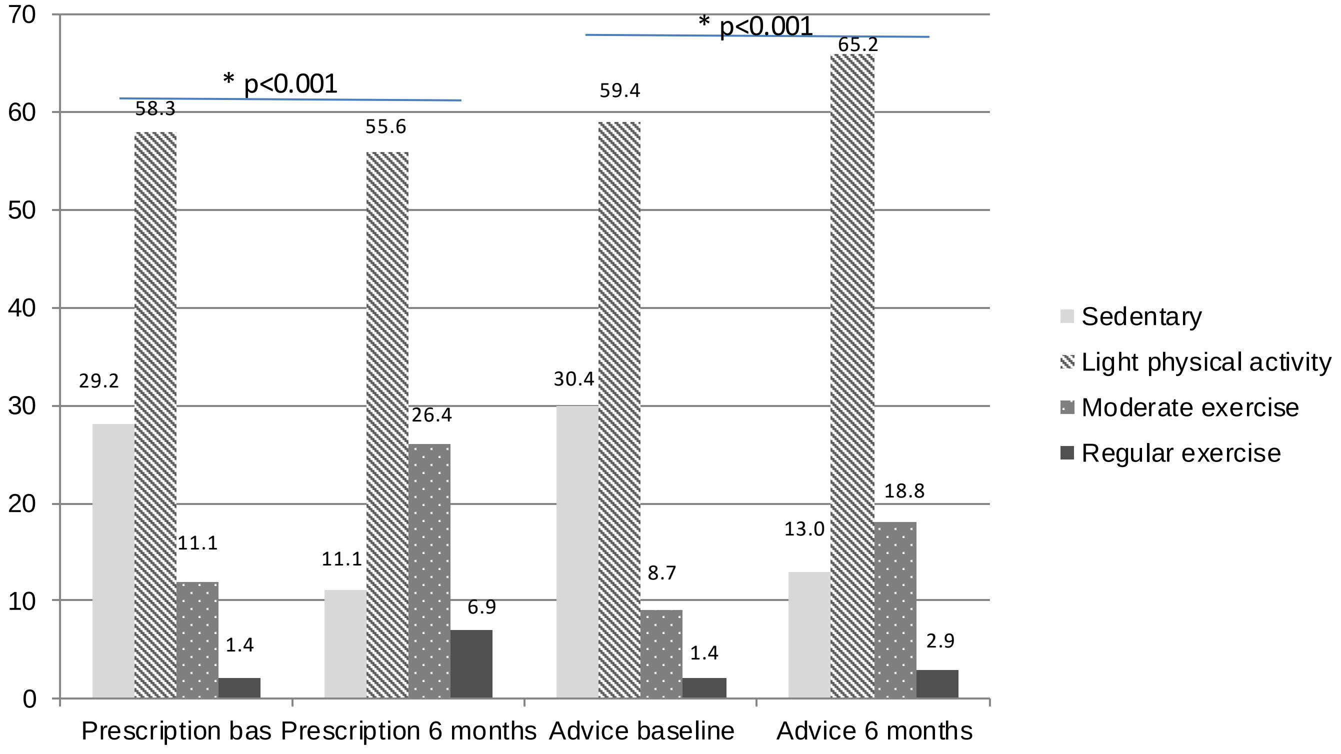

Supplement 2. Self-reported leisure time physical activity at baseline and at 6 months
Prescription group (n=72), Advice group (n=69).
Sedentary leisure time= <2h of light physical activity/week, e.g. walking, cycling.
Light physical activity during leisure time= ≥2h/week generally without sweating, e.g. walking, cykling, gardening. Moderate exercise during leisure time= 1-2 times/week, ≥30 minutes each occasion, e.g. running, swimming, tennis, or other activity that makes you sweat. Regular exercise and training during leisure time= ≥3 times a week, ≥30 minutes each time, e.g. running, swimming, tennis, aerobics, or similar exercise that makes you sweat.
* = change from baseline to 6 months in leisure time pysical activity (to more activity) was p<0.001 in both Prescription group and in Advice group.
